# Advancements in Anti-Aging Treatment Development

**DOI:** 10.3390/ijms24108515

**Published:** 2023-05-10

**Authors:** Anna Picca, Emanuele Marzetti

**Affiliations:** 1 Department of Medicine and Surgery, LUM University, 70100 Casamassima, Italy; 2 Fondazione Policlinico Universitario “A. Gemelli” IRCCS, 00168 Rome, Italy; 3 Department of Geriatrics and Orthopedics, Università Cattolica del Sacro Cuore, 00168 Rome, Italy

Aging is a complex and multifactorial process resulting, at least partly, from the generation and accrual of damage in the setting of reduced resilience [1]. A plethora of environmental and endogenous factors (i.e., exposomes) contribute to the mismatch between cellular damage and reparative capacity. Exposome constituents and their biological targets are only partially known. Nevertheless, core biological mechanisms, collectively known as the hallmarks of aging, have been identified and are actively investigated to develop therapeutics against chronic degenerative conditions [2].

Inflamm-aging refers to a chronic state of sterile, low-grade inflammation that accompanies aging, chronic diseases (e.g., cancer, diabetes, cardiovascular disease, neurodegeneration), and functional decline [3]. Inflamm-aging has been proposed as the fil rouge connecting the aging process with the pathophysiology of age-associated conditions [4]. Defective cellular quality control systems are among the candidate mechanisms underlying inflamm-aging via the accrual of intracellular “waste” (e.g., protein aggregates, damaged mitochondria, lipofuscin granules). If not appropriately disposed, cellular garbage material can be released at the systemic level and trigger inflammation [5]. Cellular senescence contributes to systemic inflammation through the release of senescence-associated secretory phenotype (SASP) factors, including cytokines, chemokines, growth and angiogenic factors, and mediators of matrix remodeling [4]. However, the mechanisms linking SASP factors, inflammation, and chronic degenerative diseases are yet to be deciphered.

The Special Issue “Advancements in Anti-Aging Treatment Development” gathers contributions by biogerontologists and geroscientists focusing on anti-aging remedies and personalized anti-inflammatory interventions to extend one’s health and lifespan [6,7,8,9,10,11,12,13].

Type 2 diabetes and Alzheimer’s disease (AD) are excellent examples of co-existing conditions, with shared pathophysiologic mechanisms that could be targeted by the same therapeutics [6]. Studies have shown that, in the setting of AD, an altered brain glucose metabolism and insulin signaling installs a state of insulin resistance (i.e., “type 3 diabetes”) that contributes to neurodegeneration and loss of synaptic plasticity [6,14]. An altered glucose metabolism has also been related to neuronal senescence, which provides a theoretical basis for exploring metabolic reprogramming as an intervention against AD [7].

Markers of metabolic derangements, together with those encompassing inflammatory, musculoskeletal, stress response, and senescence-associated pathways, have been identified among the mediators that discriminate older adults with physical frailty and sarcopenia from controls [8]. In addition, a set of molecules and mediators pertaining to energy-sensing pathways, including sirtuins, Klotho, and the mammalian target of rapamycin, have been implicated in endothelial cell senescence and vascular aging [9]. Cellular senescence has also been proposed as a therapeutic target for osteoporosis [10].

Efforts have been put forward to devise pharmacological and nutraceutical interventions to compress morbidity, allowing for an extension of disease-free lifespan [15]. Ellagic acid (EA), a phenolic compound abundant in fruits and vegetables, possesses anti-inflammatory and antioxidant properties [13]. EA protects against neurodegeneration by preserving neuronal homeostasis and viability [13]. Supplementation with Ninjin’yoeito (NYT), a mix of natural compounds used by traditional Japanese kampo medicine for treating anemia and physical weakness, was tested for its ability to alleviate oxidative stress and frailty in senescence-accelerated mouse prone 8, a murine model of senescence [12]. Significant improvements in body weight, locomotion, and walking speed were observed in mice supplemented with NYT compared with untreated controls. Lower levels of the oxidative marker 8-hydroxy-2’-deoxyguanosine were found in the muscle and brain of NYT-treated mice [12]. In addition, a lower protein abundance of cleaved caspase-3 was found in the brain tissue of mice supplemented with NYT, suggesting a possible attenuation of neuronal apoptosis [12].

Finally, genetic studies in loss-of-function (knockout) and gain-of-function (transgenic) mice have demonstrated that the CDGSH iron−sulfur domain 2 (CISD2) gene is crucial for controlling lifespan [11]. An overexpression of CISD2 improved several age-associated conditions, including AD, liver disease, and corneal epithelial health. These findings suggest that a pharmaceutically induced activation of CISD2 late in life may promote longevity. The flavonone hesperetin triggers CISD2 expression and produces anti-aging effects via the activation of genes pertaining to nitrogen, amino acid, and lipid metabolism in skeletal muscles [11].
